# Non-Conjugated Small Molecule FRET for Differentiating Monomers from Higher Molecular Weight Amyloid Beta Species

**DOI:** 10.1371/journal.pone.0019362

**Published:** 2011-04-29

**Authors:** Chongzhao Ran, Wei Zhao, Robert D. Moir, Anna Moore

**Affiliations:** 1 Molecular Imaging Laboratory, Department of Radiology, MGH/MIT/HMS Athinoula A. Martinos Center for Biomedical Imaging, Massachusetts General Hospital/Harvard Medical School, Charlestown, Massachusetts, United States of America; 2 Alzheimer's Disease Research Unit, Department of Neurology, Massachusetts General Hospital, Charlestown, Massachusetts, United States of America; University of Crete, Greece

## Abstract

**Background:**

Systematic differentiation of amyloid (Aβ) species could be important for diagnosis of Alzheimer's disease (AD). In spite of significant progress, controversies remain regarding which species are the primary contributors to the AD pathology, and which species could be used as the best biomarkers for its diagnosis. These controversies are partially caused by the lack of reliable methods to differentiate the complicated subtypes of Aβ species. Particularly, differentiation of Aβ monomers from toxic higher molecular weight species (HrMW) would be beneficial for drug screening, diagnosis, and molecular mechanism studies. However, fast and cheap methods for these specific aims are still lacking.

**Principal Findings:**

We demonstrated the feasibility of a non-conjugated FRET (Förster resonance energy transfer) technique that utilized amyloid beta (Aβ) species as intrinsic platforms for the FRET pair assembly. Mixing two structurally similar curcumin derivatives that served as the small molecule FRET pair with Aβ40 aggregates resulted in a FRET signal, while no signal was detected when using Aβ40 monomer solution. Lastly, this FRET technique enabled us to quantify the concentrations of Aβ monomers and high molecular weight species in solution.

**Significance:**

We believe that this FRET technique could potentially be used as a tool for screening for inhibitors of Aβ aggregation. We also suggest that this concept could be generalized to other misfolded proteins/peptides implicated in various pathologies including amyloid in diabetes, prion in bovine spongiform encephalopathy, tau protein in AD, and α-synuclein in Parkinson disease.

## Introduction

The amyloid β (Aβ) species of Alzheimer's disease (AD) have been considered to be an important biomarker family. In the course of AD development, Aβ monomers gradually polymerize/aggregate/cross-link into higher molecular weight species (HrMW) that include small oligomers such as dimers, tetramers, and large oligomers, profibrils, fibrils/aggregates and plaques. (Further in this manuscript we want to differentiate between HrMW that include all species higher than monomers and high molecular weight speciaes (HMW) that include only large oligomers, profibrils, fibrils/aggregates and plaques). Controversies remain regarding which Aβ species are the primary contributors to the AD pathology [1], [2] and which species could be used as the best biomarkers for its diagnosis. However, numerous studies suggest that all HrMW species are neurotoxic [3], [4], [5], [6]. Identifying potential therapeutics preventing monomeric Aβ species from transforming into higher molecular weight species would be extremely beneficial for AD patients. Nonetheless, drug screening for inhibitors of Aβ aggregating is largely dependent on Thioflavin assays [7], [8], [10]. It is well known that Thioflavin T is only effective in monitoring the formation of HMW species that include large oligomers, profibrils, fibrils/aggregates and plaques [7], [9], [10], [11] but not suitable for small oligomers that are also toxic [7], [12]. Therefore, a cheap, efficient and quantitative method capable of monitoring the aggregation from monomers to any HrMW species is highly desirable [13].

**Figure 1:**
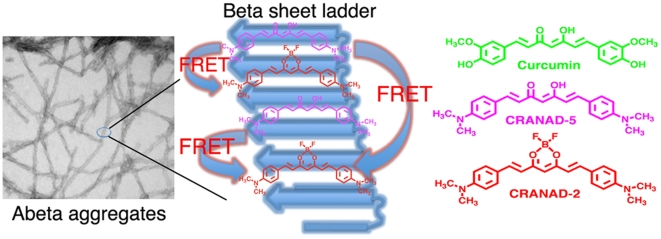
The principle of non-conjugated small molecular FRET. Left: Mixing non-conjugated FRET partner molecules with the Aβ aggregates/fibrils, which consist of numbers of β-sheet ladders, has a high probability of incorporating the partner into the Aβ species within 10 nm proximity to generate a viable FRET signal. Right: Chemical structures of curcumin, CRANAD-2 and CRANAD-5.

FRET (Foster Resonance Energy Transfer) is a distance-dependent energy transfer between two chromophores [14]. In practice, proximity of less than 10 nm between donor and acceptor and a substantial overlap of donor emission and acceptor excitation spectra are essential for an efficient FRET pair [15]. The intrinsic structure of Aβ aggregates/plaques represents a large number of compacted ladders of β sheets containing Aβ40/42 peptide [16], [17]. Numerous small molecules could be retained in the aggregates/plaques/oligomers, provided that they are capable of intercalating into the β sheets. For example, the insertion of the molecule curcumin into these β sheets has been reported [18]. We reasoned that two non-conjugated FRET partner molecules with the affinity for β sheets would, upon mixing with the Aβ species (aggregates, fibrils and plaques), have a high probability of randomly incorporating themselves into the Aβ species within10 nm proximity to generate a viable FRET signal (Fig. 1).

In this report, we first demonstrated that Aβ aggregates could be used for assisting the assembly of a FRET pair consisting of two non-conjugated curcumin analogues, and then we extended this concept to Aβ dimers. Lastly, we demonstrated the feasibility of differentiation of monomers from HrMW species. We believe that the described method bears a potential in AD research for drug screening and possible disease staging.

## Results

First, we tested our non-conjugated small molecule FRET concept by using Aβ40 aggregates as the assembling platform, and CRANAD-2 and CRANAD-5 as the small molecule FRET pair. Aβ40 aggregates were prepared as described (Material and Methods) and the morphological structure was confirmed by TEM (Fig. S1). We have previously shown that CRANAD-2 and CRANAD-5, analogues of curcumin, were specific fluorescence imaging probes for Aβ species ([19] and data not shown). Structurally, CRANAD-2 and CRANAD-5 have a similar backbone, indicating that the two molecules may have an analogous mechanism of interaction with Aβ aggregates (Fig. 1). Moreover, CRANAD-5′s emission and CRANAD-2′s excitation have a reasonable spectral overlap (Fig. S2 lower panel), which spans from 550 nm to 700 nm. Based on these facts, we reasoned that CRANAD-5/CRANAD-2 could be a potential FRET pair for Aβ aggregates. Furthermore, the apparent binding affinity of CRANAD-5 (Kd = 10 nM) was very close to that of CRANAD-2 (Kd = 38 nM), indicating that both probes would integrate within Aβ species with similar probabilities. Collectively, these facts formed the basis for further testing of CRANAD-5/CRANAD-2 as a possible in vitro FRET pair.

**Figure 2:**
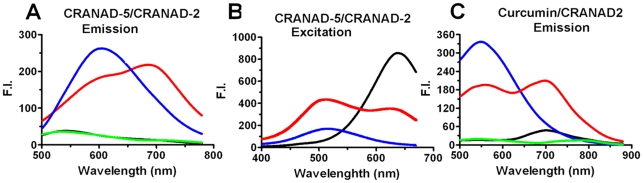
Validation of FRET phenomenon in solution with Aβ40 aggregates. (A) Spectra overlay showing FRET phenomenon in a solution of CRANAD-5 and CRANAD-2 with Aβ40 aggregates (a) (red); spectrum of control solution of CRANAD-5 with Aβ40 aggregates (b) (blue); spectrum of control solution of CRANAD-2 with Aβ40 aggregates (c) (black, overlapped with the green line); spectrum of control solution of CRANAD-5 and CRANAD-2 without Aβ40 aggregates (d) (green, overlapped with the black line). (B) Excitation spectra overlay of solution (a) (red), (b) (blue) and (c) (black) with the emission at 700 nm. Note two excitation peaks for solution (a). (C) Spectra overlay of solutions of curcumin and CRANAD-2 with Aβ40 aggregates: FRET (red), spectrum of control solution of curcumin with Aβ40 aggregates (blue), spectrum of control solution of CRANAD-2 with Aβ40 aggregates (black), and spectrum of control solution of curcumin and CRANAD-2 without Aβ40 aggregates (green).

To this end, we examined four solutions: (a) CRANAD-5 and CRANAD-2 with Aβ40 aggregates; (b) CRANAD-5 with Aβ40 aggregates; (c) CRANAD-2 with Aβ40 aggregates; and (d) CRANAD-5 and CRANAD-2 without Aβ40 aggregates. In our search for the best parameters resulting in the highest FRET peak resolution we measured the spectra of the four solutions (a–d) with excitation at 400 nm, 420 nm, 480 nm, 500 nm, 540 nm, and 580 nm. At 420 nm excitation we observed unambiguous FRET phenomenon with the best resolution for the peak at 700 nm (the acceptor's emission peak) (Fig. 2A). At this excitation, the intensity of (a) was 30% lower than that of (b) at 610 nm (the peak emission of the donor CRANAD-5) but it was 17-fold higher than that of (c) at 700 nm, the peak emission of the acceptor CRANAD-2, which could not be excited without its FRET partner CRANAD-5 at 420 nm excitation. There was no apparent FRET signal for solution (d) because of the absence of Aβ40 aggregates. In solution (a), there are two states for this non-conjugated FRET pair, i.e. CRANAD-2/5 paired within the FRET distance and CRANAD-2 or -5 bound to the beta-sheets of the aggregates, though not within the efficient FRET range. Therefore, the fluorescence intensity (F.I.) signal at 700 nm is a sum of the FRET signal and non-FRET signal from excitation of CRANAD-2 and CRANAD-5 at 420 nm. Since the signal from CRANAD-2 was not significant (Fig. 2A, black line, overlapped with green line), we could approximately estimate the respective contributions by linear spectral unmixing using linear programming (details are described in Text S1 and Fig. S3). The contribution of the non-FRET signal of CRANAD-5 was about 44.8, and the actual FRET signal at 700 nm was 156.4.

We also examined the concentration ratio between CRANAD-5 and CRANAD-2, and found that once it was close to 1∶1, the intensity almost plateaued (Fig. S4). In addition, the excitation spectra of the above solutions (a–c) were recorded by setting the emission at 700 nm. We found two peaks for solution (a) that corresponded to the excitation peaks of CRANAD-5 and CRANAD-2, indicating an obvious FRET partnership between these two molecules (Fig. 2B). When tested whether curcumin and its analogues could form a FRET pair for the aggregates, we found that the pair of curcumin/CRANAD-2 was able to generate a FRET signal as well (Fig. 2C).

**Figure 3:**
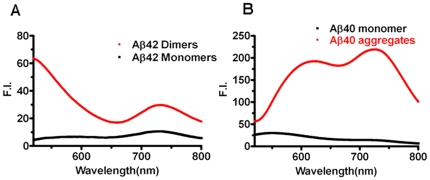
Differentiation of Aβ monomers from higher molecular weight Aβ species, which include aggregates/fibrils, and dimers. (A) Spectra overlay of Aβ42 dimers (red) and Aβ42 monomers (black). (B) Spectra overlay of Aβ40 aggregates (red) and Aβ40 monomers (black).

To test whether dimers could be used to assemble CRANAD-5/CRANAD-2 into a FRET pair, we incubated CAPS Aβ42 dimers with the FRET partner. The Aβ42 dimers were prepared as reported [20], purified by HPLC and characterized by Western blot. The TEM image indicated that the preparation did not contain significant deposits (Fig. S5). Additionally, there was no significant fluorescence intensity change with a Thioflavin T test (data not shown). As expected, we observed the apparent FRET signal (Fig. 3A) from the Aβ42 dimers solution, indicating that these species could be used as an intrinsic FRET platform.

To test whether Aβ monomers could be used to assist the FRET pair in assembly, we incubated Aβ40 monomers with a CRANAD-5/-2 pair. The Aβ40 monomers were prepared from HFIP (hexafluoroisopropanol) treated solution [21], and purified by HPLC (see Material and Methods). The monomeric state was characterized by SDS-Page gel, and the morphology was confirmed by TEM (Fig. S5). As we expected, no significant FRET signal was observed at 700 nm (Fig. 3B), indicating that these monomers do not form a FRET platform. In addition, no significant fluorescence intensity change was observed with a Thioflavin T test (data not shown). Similar results were obtained with Aβ42 monomers (Fig. 3A). Because the CRANAD-5/-2 FRET pair could be assembled with the assistance of HrMW species such as dimers and aggregates, but not the monomers, we suggested that the non-conjugated FRET technique could be used to differentiate monomers from higher molecular weight Aβ species, such as dimers, oligomers, and aggregates.

For a given solution, we could use this method to calculate the concentrations of monomers (C_(mono)_) and HrMW species (C_(hrmw)_). The FRET signal at 700 nm was proportional to the concentration of higher molecular weight species, i.e. 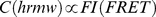
(1)


**Figure 4:**
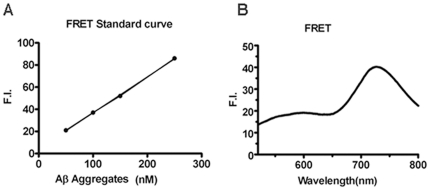
Testing with commercial available Aβ40 peptide. (A) Standard curve of FRET with Aβ40 aggregates. (B) Fluorescence spectra of Aβ40 sample from rPeptide with CRANAD-5/-2 FRET pair.



Therefore, based on the FRET signal we could generate a standard curve (Fig. 4A) described in Eq.(2),

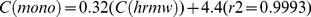
(2)


If the Aβ species solution is a synthetic peptide solution, the total Aβ species consist of monomers and HrMW species, therefore,
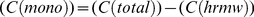
(3)


We tested a sample of 250 nM Aβ40 peptide from rPeptide (Cat. No. A-1001-2 (TFA)) by using a FRET (Fig. 4B). Using the obtained fluorescence intensity value and the calibration curve (Equation 2), we calculated the concentration of high molecular weight species (C_(hrmw)_) = 102.8 nM. Next, from Equation (3) we obtained the concentration of monomeric species (C_(mono)_) = 147.2 nM.

Due to the complicated nature of HrMW species and their possible influence on FRET we realize that the obtained data could only serve as the first approximation. However, we believe that this is an important first step in identifying the amount of various species present during AD progression.

## Discussion

We demonstrated that high molecular weight Aβ species could be used as intrinsic platforms for assembling a non-conjugated non-bioengineered FRET pair. These pairs can be used for differentiating monomers from HrMW species, which have neurotoxic effects. Differentiating Aβ monomers from potentially damaging species bears a potential for Alzheimer's disease early detection and treatment monitoring. Although the exact normal physiological role for Aβ monomers remains unknown, they have been shown to serve as ligands for a number of different receptors and other molecules [22]. Recently it has been indicated that Aβ monomeric peptides could be involved in the antimicrobial function of the innate immune system [23]. Meanwhile, aggregation of Aβ monomers is probably one of the most important initial points in AD progression. A screening method to seek compounds capable of keeping Aβ at the monomeric status is highly desirable. We believe our approach could be used as an inexpensive, efficient, and easy-to-use tool for monitoring the aggregating stage from monomers to oligomers.

Currently, for biofluids with low Aβ concentrations such as CSF and cell culture medium, ELISA tests with anti-Aβ antibodies are used for quantification of concentrations of monomers, dimers, and other HrMW species. However, this method is expensive, time-consuming, and inconsistent within different operators and testing centers [3], [4], [5], [6]. For high concentration samples, CD (circular dichroism) and QLS (quasielastic light scattering spectroscopy) have been used to monitor the progression of aggregation from monomers to HrMS species, however, these methods are impractical for routine clinical sample analysis [11], [24], [25]. We believe our approach would be a useful complementary tool for the above-mentioned methods. The advantages of our method include low costs, fast reaction time, good sensitivity, and straightforward protocol.

**Figure 5:**
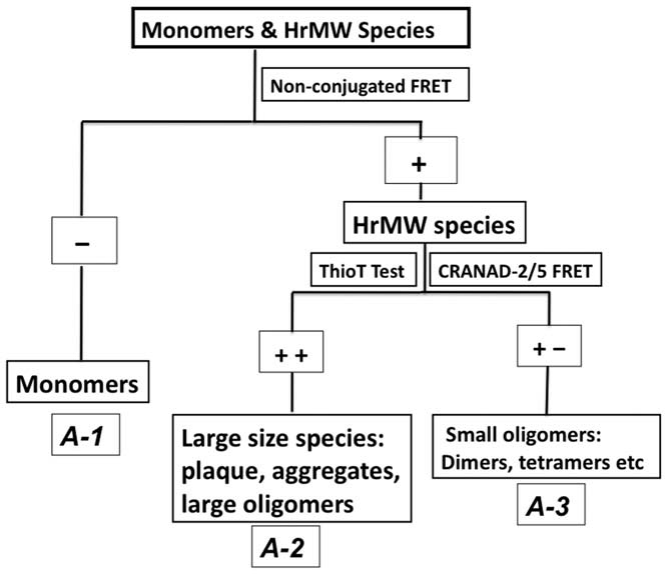
Flow chart and principle for differentiating various Aβ species based on non-conjugated FRET technique. (A-1) Differentiating Aβ monomers from high molecular weight species; (A-2, A-3) differentiating large size species (A-2) from small oligomers (A-3).

It is important to note that the proposed FRET platform is not only able to differentiate monomers from HrMW species (Fig. 5 A-1), but is also able to differentiate small oligomers from the large size species (plaques, fibrils, profibrils and large oligomers) when it is combined with a Thioflavin T test (Fig. 5). Thioflavin T has been used widely to detect plaques in brain tissues and fibrils/aggregates/profibrils in solutions [7], [8], [10]. It has been recently reported that it is also capable of detecting soluble oligomers [9]. On the other hand, Biancalana et al has predicted that 4-5 beta sheets are needed for Thioflavin T binding [7], [12], indicating that it can not detect small oligomers such as dimers and tetramers, which have been reported as highly toxic species [26], [27]. We envision that the proposed flow chart in Fig. 5 could be used to differentiate various Aβ species. Since our FRET is able to detect HrMW species that include large size species and small oligomers, while Thiofalvin T is capable of detecting large size species, for a given pure solution, if the testing results with Thioflavin T and FRET are both positive (Fig. 5 A-2), the solution could contain large size species. If a FRET test is positive and a Thioflavin T test is negative (Fig. 5 A-3), the solution could contain small oligomers. In practice, we could generate two standard curves for Thioflavin T and FRET respectively, to estimate the concentrations of large size species, small oligomers and monmers.

The FRET pair described in this study served only as a proof-of-concept. To achieve higher sensitivity and better selectivity, we are currently in the process of developing a small compound library to seek better matching pairs.

Lastly, we suggest that the proposed concept could be generalized to other misfolding proteins/peptides such as amylin in diabetes, prion in mad cow disease, tau protein in AD, and α-synuclein in Parkinson disease if specific FRET matched fluorophore pairs are found. We also envision that this technique could be extended to studies of protein/protein and protein/RNA interactions with fluorophore tagged ligands.

## Materials and Methods

Reagents used for synthesis were purchased from Aldrich and used without further purification. Column chromatography was performed on silica gel slurry (SiliCycle Inc., 60 Å, 40-63 mm) packed into glass columns. Synthetic amyloid-β peptide (1-40) was purchased from rPeptide (Bogart, GA, 30622) and aggregates for *in vitro* studies were generated by slow stirring for 3 days in PBS buffer at room temperature. ^1^H and ^13^C NMR spectra were recorded at 500 MHz and 125 MHz respectively, and reported in ppm downfield from tetramethylsilane. Mass spectra were obtained at Harvard University, at the Department of Chemistry Instrumentation Facility.

### Synthesis of CRANAD-2 and CRANAD-5

#### CRANAD-2

The synthesis was performed according to our previously reported procedure [19].

#### CRANAD-5

The synthesis followed the reported procedure with some modifications [28]. Boric oxide (700.0 mg, 10.0 mmol) was dissolved in DMF (10.0 mL) at 120°C. Ensuring that most of the boric oxide was dissolved was crucial for high yield. To this solution, acetylacetone (1.1 mL, 10.0 mmol) was added, followed by tributyl borate (5.4 mL, 20.0 mmol) at 120°C and stirred for 5 min. To the borate complex, 4-*N,N'*-dimethylamino-benzaldehyde (3.1 g, 20.0 mmol) was added and stirred for 5 min. A mixture of 1,2,3,4-tetrahydroquinoline (0.2 mL) and acetic acid (0.4 mL) in DMF (4.0 mL) was added to the reaction mixture and heated to 120°C for 2 h. After cooling to room temperature, the reaction mixture was poured into ice water (500 mL), and a reddish precipitate was collected. The precipitate was further purified with a silica gel column using ethyl acetate/hexanes (50∶50) as eluent to yield the reddish powder of CRANAD-5 (1.8 g, 52.7 %). ^1^H NMR (CDCl_3_) δ(ppm) 3.03 (s, 6H), 5.73 (s, 1H), 6.42 (d, 2H, J = 16.0 Hz), 6.68 (d, 4H, J = 10.0 Hz), 7.45 (d, 4H, J = 10.0 Hz), 7.60 (d, 2H, J = 16.0 Hz); ^13^C NMR (CDCl_3_) δ(ppm) 40.4, 101.1, 112.1, 119.4, 123.3, 130.0, 140.8, 151.8, 183.6. The spectra were consistent with the reported value [28].

#### Aβ40 aggregates preparation

Aβ40 peptide (1.0 mg) powder was suspended in 1% ammonia hydroxyl solution (1.0 mL), and 0.1 mL of the resulting solution was diluted tenfold with PBS buffer (pH 7.4) (final concentration 25 µM) and stirred at room temperature for 3 days. TEM was performed using a JEOL 1011 electron microscope. Ten microliters of the above Aβ40 peptide solution was mounted on a *formava*-coated copper grid for 5 minutes, and the grid was dried off with filter paper. Then, 10 µl of 2**%** aqueous phosphotungstic acid (adjust pH to 7.3 using 1N NaOH) was dropped immediately onto the grid, and left for 30 seconds. The grid was placed directly into the grid box and allowed to air-dry for several hours before observation. The images confirming the formation of aggregates were taken at 80.0 kv with direct magnification of 10,000 (Fig. S1). This solution was kept at 4°C for storage.

#### Aβ40/42 monomers preparation

Aβ40/42 monomers were prepared by further purification of commercially available Aβ40/42 peptide (rPeptide, catalogue No. A-1153-1 and A-1163-1 with HFIP treatment) using HPLC. The purified monomers were stored as powder/film. Before use, the peptides were dissolved in hexafluoroisopropanol (HFIP) as stock solutions [21]. SDS-gel testing of monomeric Aβ40/42 peptides was performed on a 4–20% gradient Tris gel (Bio-Rad) and SeeBlue®plus2 (Invitrogen)(4–250 KD) was used as a molecular weight marker. A ten microliter sample (25 µM) was loaded and MES buffer was used for running the gel. Both SDS-GEL and TEM results confirmed that the products were not oligomerized or aggregated (data shown in Fig. S5).

#### Aβ42 dimers preparation

Aβ42 dimers were prepared by following the reported procedure [20] (the detailed procedure is described in Text S1). Western blot for CAPS Aβ42 dimers and Aβ42 monomers was performed using monoclonal antibody mAb 6E10 raised against residues 1–17 of the Aβ peptide (Calbiochem) (data shown in Fig. S5).

#### Binding constant measurements

To PBS solutions (1.0 mL) of Aβ40 aggregates (2.5 µM, calculation based on Aβ40 peptide concentration), various amounts of CRANAD-5 were added to final concentrations of 5.0, 10.0, 20.0, 40.0, 100.0, 200.0 nM, and their fluorescence intensities at 610 nm were recorded (Ex: 520 nm). The Kd binding curve was generated using Prism 5.0 software with nonlinear one-site binding regression to give Kd = 10.5±2.5 nM.

#### Spectral overlap measurements

Emission spectra were recorded for a solution of curcumin (0.5 µM) and Aβ40 aggregates (1.0 µM) in PBS (1.0 mL) (Ex: 470 nm, Ex slit = 10 nm, and Em slit = 10 nm), and normalized to the highest reading of 1.0. For the excitation spectrum of CRANAD-2, a solution of CRANAD-2 (0.5 µM) and Aβ40 aggregates (1.0 µM) in PBS (1.0 mL) were used (Em: 800 nm, Ex slit = 10 nm, and Em slit = 10 nm). The recorded spectrum was normalized to the highest reading of 1.0. The final spectral overlap was generated using Prism 5.0 software.

#### FRET

A solution of Aβ40 aggregates (1.0 µM) was mixed with 0.5 µM of CRANAD-2, and incubated at room temperature for 1 min, following by the addition of 0.5 µM of CRANAD-5 (a). The FRET spectrum of the resulted solution was measured within 1 min (Ex: 420 nm, Ex slit = 10 nm, and Em slit = 10 nm). As controls, a solution of 0.5 µM CRANAD-5 with Aβ40 aggregates (1.0 µM) (b), a solution of 0.5 µM CRANAD-2 with Aβ40 aggregates (1.0 µM) (c) and a solution of 0.5 µM CRANAD-5 and 0.5 µM CRANAD-2 without aggregates (d) were used.

A similar procedure was used for recording the FRET spectrum of the curcumin/CRANAD-2 pair (Ex: 470 nm, Ex slit = 10 nm, and Em slit = 10 nm). For excitation spectra recording, Em = 700 nm was used, with 10 nm Em/Ex slits.

#### Standard curve of FRET

Fluorescence spectra of CRANAD-5/-2 FRET pair (250 nM for both compounds) with 50, 100, 150, 250 nM Aβ40 aggregates were recorded. The standard curve was plotted as Aβ40 aggregates concentration vs. FI at 700 nm, and fitted using linear regression.

## Supporting Information

Figure S1 TEM image of the Aβ40 aggregates.
(PNG)

Figure S2 Upper: Excitation and emission spectra of CRANAD-5 (with Abeta40 aggregates); Middle: Excitation and emission spectra of CRANAD-2 (with Abeta40 aggregates); and Lower: Spectral overlap of the emission of CRANAD-5 and the excitation of CRNAD-2.
(PNG)

Figure S3 Approximate estimation of the actual FRET signal by linear spectral unmixing. Red line is the measured FRET spectrum; green line is the unmixed spectrum for CRANAD-5 with Aβ40 aggregates; blue line is the unmixed spectrum for actual FRET spectrum without contamination from the non-FRET signal of CRANAD-5.
(PNG)

Figure S4 The titration curve of CRANAD-2 (250 nM) with various concentrations of CRANAD-5.
(PNG)

Figure S5 (A) SDS-Page gel of Abeta40 and Abeta42 monomers; (B) TEM image of Abeta40 monomers (negative staining with PTA); (C) Western-blot of Abeta42 dimers; (D) TEM image of Abeta42 dimers. Scale bar: 100 nm.
(PNG)

Text S1 Brief description of Preparation of dimeric Aβ fractions and Spectral unmixing method.
(DOC)
