# Supplementary figures and images for: Non-Conjugated Small Molecule FRET for Differentiating Monomers from Higher Molecular Weight Amyloid Beta Species
Source: PLoS One. 2011 Apr 29;6(4):e19362. doi: 10.1371/journal.pone.0019362 (PMC3084834; doi:10.1371/journal.pone.0019362)

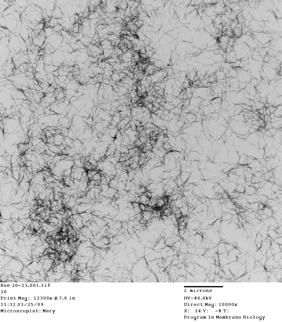

Supplement: Figure S1 — TEM image of the Aβ40 aggregates. (PNG) [file pone.0019362.s001.png]

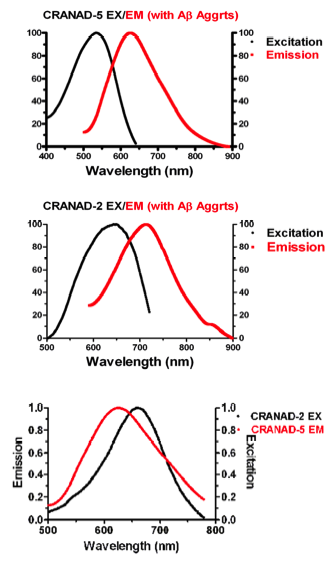

Supplement: Figure S2 — Upper: Excitation and emission spectra of CRANAD-5 (with Abeta40 aggregates); Middle: Excitation and emission spectra of CRANAD-2 (with Abeta40 aggregates); and Lower: Spectral overlap of the emission of CRANAD-5 and the excitation of CRNAD-2. (PNG) [file pone.0019362.s002.png]

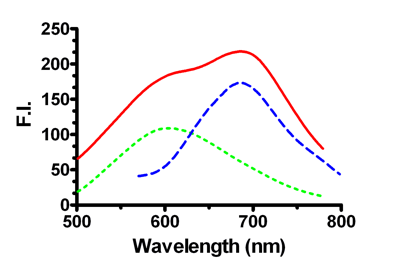

Supplement: Figure S3 — Approximate estimation of the actual FRET signal by linear spectral unmixing. Red line is the measured FRET spectrum; green line is the unmixed spectrum for CRANAD-5 with Aβ40 aggregates; blue line is the unmixed spectrum for actual FRET spectrum without contamination from the non-FRET signal of CRANAD-5. (PNG) [file pone.0019362.s003.png]

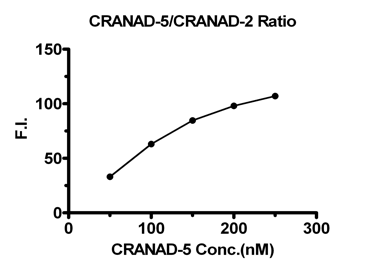

Supplement: Figure S4 — The titration curve of CRANAD-2 (250 nM) with various concentrations of CRANAD-5. (PNG) [file pone.0019362.s004.png]

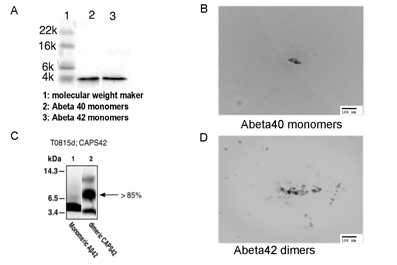

Supplement: Figure S5 — (A) SDS-Page gel of Abeta40 and Abeta42 monomers; (B) TEM image of Abeta40 monomers (negative staining with PTA); (C) Western-blot of Abeta42 dimers; (D) TEM image of Abeta42 dimers. Scale bar: 100 nm. (PNG) [file pone.0019362.s005.png]
