# Supplementary material for: Non-Conjugated Small Molecule FRET for Differentiating Monomers from Higher Molecular Weight Amyloid Beta Species
Source: PLoS One. 2011 Apr 29;6(4):e19362. doi: 10.1371/journal.pone.0019362 (PMC3084834; doi:10.1371/journal.pone.0019362)
Supplement: Text S1 — Brief description of Preparation of dimeric Aβ fractions and Spectral unmixing method. (DOC) [file pone.0019362.s006.doc]

**Non-conjugated small molecule FRET and its potential application for differentiating amyloid beta species**

Chongzhao Ran, Wei Zhao, Robert D. Moir and Anna Moore[[1]](#footnote-2)

*Molecular Imaging Laboratory, MGH/MIT/HMS Athinoula A. Martinos Center for Biomedical Imaging, Department of Radiology, Massachusetts General Hospital/Harvard Medical School Charlestown, Boston, Massachusetts 02129*

**Preparation of dimeric Aβ fractions:**

In our experiments, we used Aβ1-40 (Aβ40) and Aβ1-42 (Aβ42) that was prepared and purified by Dr. James I. Elliott at Yale University (New Haven, CT), using solid-phase peptide synthesis. Bulk powdered peptides were first dissolved in 30 % trifluoroethanol (TFE) at 1 mg/ml. Five hundred microliter aliquots of the stock solutions were lyophilized and stored under nitrogen at -20°C. Stock solutions at 2 mg/ml were prepared the day of experimentation from the peptide films by solubilizing a second time in 20 mM NaOH. Sodium hydroxide stock peptide solutions were neutralized by dilution in PBS immediately before use. Peptide concentrations in stock solutions were determined by bicinchoninic acid (BCA) protein assay. The validity of BCA for assaying Ab peptides has been established previously (1, 2).

Stock Aβ solution was diluted to 200 µg/ml in PBS containing 200 µM ascorbic acid and 25 µM CuCl_2_ and incubated at 37°C for 2 hours. Incubants were then diluted in 5 % 2,2,2-trifluoroethanol (TFE) and 0.1 % trifluoroacetic acid (TFA) before being loaded onto a C18 reverse phase HPLC column. The column was developed with an increasing (5-100%) gradient of TFE. Aqueous and TFE buffers contained 0.05% TFA and all chromatographic procedures were at 80°C and at a flow rate of 1ml/min. Elute fractions were assayed by mAb 6E10 immunoblot and dimer enriched fractions were pooled, lyophilized, and stored at -70°C.

**Spectral unmixing method (for Fig. S3):**

First we recorded the fluorescence spectra of solutions of (a) (CRANAD-2 with Abeta aggregates), (b) (CRANAD-5 with aggregates), and (c) (mixture of CRANAD-2 and CRANAD-5 with aggregates (FRET solution)).

Principally the signal of (c) solution (F*_(c)_*) (the observed FRET signal) is the sum of actual FRET signal and non-FRET signal from the donor (the non-FRET contribution of the acceptor is insignificant), therefore (F*_(c)_*) is proportional to the linear sums of signal of (a) solution (F*_(a)_*) and (b) solution (F*_(b)_*):

---- Eq. (1)

K_1_and K_2_ are the proportional constants for the contributions to the observed FRET signal (F*_(c)_*), and c_1_ and c_2_ are adjusting constants. By using YALMIP toolbox in MATLAB to conduct linear programming, we were able to fit the spectral curve of (c) solution (the observed FRET) into the following equation and the residual error was within 6% of (F*_(c)_*):

---- Eq. (2)

From the above Eq. (2), we could unmix the observed FRET signal (F*_(c)_*) into two individual spectral signals (F*_(unmix1)_*) and (F*_(unmix2)_*), where (F*_(unmix1)_*) is the unmixed FRET (the actual FRET) signal, and (F*_(unmix2)_*) is the non-FRET contribution from the donor. (F*_(unmix1)_*) and (F*_(unmix2)_*) could be described as:

**Supplemental Figure Legend**

**Fig. S1** TEM image of the Aβ40 aggregates.

**Fig. S2** Upper: Excitation and emission spectra of CRANAD-5 (with Abeta40 aggregates); Middle: Excitation and emission spectra of CRANAD-2 (with Abeta40 aggregates); Lower: Spectral overlap of the emission of CRANAD-5 and the excitation of CRNAD-2.

**Fig. S3** Approximate estimation of the actual FRET signal by linear spectral unmixing. Red line is the measured FRET spectrum; green line is the unmixed spectrum for CRANAD-5 with Aβ40 aggregates; blue line is the unmixed spectrum for actual FRET spectrum without contamination from the non-FRET signal of CRANAD-5.

**Fig. S4** The titration curve of CRANAD-2 (250nM) with various concentrations of CRANAD-5.

**Fig. S5** A: SDS-Page gel of Abeta40 and Abeta42 monomers; B: TEM image of Abeta40 monomers (negative staining with PTA); C: Western-blot of Abeta42 dimers; D: TEM image of Abeta42 dimers. Scale bar: 100 nm.

1. Correspondence should be addressed to:

   Anna Moore, Ph.D., Molecular Imaging Laboratory

   MGH/MIT/HMS Athinoula A. Martinos Center for Biomedical Imaging, Department of Radiology

   Massachusetts General Hospital/Harvard Medical School

   Bldg.75, 13th St.

   Charlestown, Massachusetts 02129

   Tel:(617) 724-0540

   Fax:(617) 643-4865? [↑](#footnote-ref-2)
